# Dietary Advanced Glycation End products interacting with the intestinal epithelium: What do we really know?

**DOI:** 10.1016/j.molmet.2023.101734

**Published:** 2023-04-29

**Authors:** Fleur A.C. Jansen, Vincenzo Fogliano, Josep Rubert, Tamara Hoppenbrouwers

**Affiliations:** 1 Department of Food Quality and Design, Wageningen University and Research, Bornse Weilanden 9, 6708 WG, Wageningen, the Netherlands; 2 Nutritional Biology, Division of Human Nutrition, Wageningen University and Research, Stippeneng 4, 6708 WE, Wageningen, the Netherlands; 3 Food and Biobased Research, Wageningen University and Research, Bornse Weilanden 9, 6708 WG, Wageningen, the Netherlands

**Keywords:** Dietary advanced glycation end products, Intestinal epithelium, In vitro, In vivo, Methodology

## Abstract

**Background:**

Advanced Glycation End products (AGEs) are a heterogeneous group of stable reaction products formed when amino acids, peptides, or proteins are glycated by the non-enzymatic Maillard Reaction. The formation and accumulation of these products *in vivo* are linked to many inflammation-based pathological outcomes and part of the pathophysiology of non-communicable diseases like eye cataracts and Alzheimer's disease. Since our diet contains high levels of the same compounds, it has been questioned whether their consumption is also detrimental to health. However, this is still under debate. In this context, the intestinal epithelium is an important target tissue since it is chronically exposed to relatively high concentrations of dietary AGEs.

**Scope of review:**

This review summarizes the current evidence on the impact of dietary AGEs on the intestinal epithelium and critically reflects on its methodology.

**Major conclusions:**

In healthy rodent models, an inflammation-independent impaired intestinal barrier function is claimed; however, dietary AGEs showed anti-inflammatory activity in IBD models. *In vitro* studies could be a valuable tool to unravel the underlying mechanisms of these effects, however the available studies face some limitations, e.g. lack of the physicochemical characterization of the glycated proteins, the inclusion of the proper controls and the dose-dependency of the effect. In addition, studies using more advanced *in vitro* models like intestinal organoids and co-cultures with immune cells exposed to gut microbial metabolites derived from the fermentation of AGEs are still needed.

## 1 Introduction

Advanced Glycation End products (AGEs) are a heterogeneous group of stable reaction products from the Maillard Reaction. This reaction is a non-enzymatic process in which simple sugars (e.g. glucose) react with free amino groups leading to glycation of amino acids, peptides, and proteins, i.e. covalent bonding between the sugar molecules and free amino groups [1]. In the case of AGEs, the reacting amines are part of lysine or arginine residues. Well-known lysine-based AGEs are carboxymethyllysine (CML) and carboxyethyllysine (CEL), while a frequently studied arginine-based AGE is methylglyoxal-derived hydroimidazolone 1 (MG-H1). In addition to this characteristic, AGEs can be distinguished based on whether their formation facilitates protein cross-linking. For example, the AGE pentosidine acts as a covalent cross-link between lysine and arginine residues [2]. Since the Maillard Reaction is a non-enzymatic process driven by temperature, AGEs are formed endogenously (*in vivo*) as well as exogenously (food processing), admittedly at different rates [3].

## 2 Endogenous vs exogenous AGEs

Although their chemical structures are similar, endogenous and exogenous AGEs were approached differently in research. Where endogenously formed AGEs are investigated for their role in the pathophysiology of several non-communicable diseases, exogenously formed AGEs have mainly been investigated in relation to palatability and protein digestibility. However, recently, scientist started questioning whether the consumption of these AGEs may also contribute to several diseases generating an ample debate [[3], [4], [5], [6]].

Endogenously, the Maillard Reaction causes body protein misfolding and aggregation, respectively, dependent on whether the formation occurs within the same protein or between different proteins [3]. Although these proteins become dysfunctional, they are not always cleared properly. This accumulation of misfolded and aggregated proteins contributes to several non-communicable age-related diseases, such as cataracts, Alzheimer's disease, and atherosclerosis [[7], [8], [9]]. In addition, endogenous AGEs are described to activate the Receptor for Advanced Glycation End products (RAGE). Upon RAGE induction, a variety of intracellular signaling pathways have been described to be activated, among which pathways involved in cell survival and apoptosis (e.g. ERK1/2, PI3K/Akt, Caspase 8) and inflammation (NF-kB, JNK) [10]. Together, endogenous AGEs are considered to be involved in disease pathology by generating dysfunctional proteins and inducing pro-inflammatory signaling.

Exogenously, the Maillard Reaction is essential for developing the food's color, taste, and odor while cooking. Especially high-temperature processed low-moisture foods such as bakery products have been described to contain high levels of these dietary AGEs [3,11]. Much research has been performed on their effect on protein digestibility and, subsequently, the bioavailability of amino acids. *In vitro* research shows that the digestibility of food proteins decreases with the level of glycation since aggregates are formed and lysine and arginine would no longer be available for cleavage by pepsins. As a result, these studies suggest that free AGEs and dipeptides can be considered (partially) bioavailable, while large glycated peptides and proteins would hardly be bioavailable [[12], [13], [14]]. However, reduced bioavailability of amino acids from glycated proteins was not observed in a clinical setting, except for lysine [15]. The latter could also be related to glycation-induced structural changes interfering with the employed detection method, leading to an underestimation of the amount of absorbed lysine. In another study, Van Dongen et al. [16] investigated the digestibility of glycated proteins and bioavailability of protein-bound AGEs in mice. Mice were fed a baked chow diet, containing more protein-bound CML but less free CML than the standard chow diet. The authors observed increased levels of free and protein-bound CML in plasma. On top of that, the authors also quantified other AGEs (CEL and MG-H1) in different tissues (liver and kidney). Overall, the results were inconsistent. For example, free MG-H1 was elevated in plasma, liver, and kidney, but protein-bound MG-H1 did not increase. By contrast, CEL was not elevated in the liver. This suggests tissue-dependent effects and discrepancies between various AGEs. The increased levels of reactive oxoaldehydes in the baked chow diet could be a confounding factor since they may promote endogenous formation of AGEs. Interestingly, all effects observed in this study were shown to be reversible by switching back to a normal chow diet. To sum up, *in vitro* research shows decreased digestibility of glycated proteins, but *in vivo* studies suggest that the bioavailability of (modified) amino acids from these glycated proteins is not hampered. However, more research is required in this respect.

Next to the bioavailability of dietary AGEs, many researchers also focused on the potential systemic health effects they exert after being absorbed. For example, in the mice study discussed before [16], the authors also measured inflammatory markers in plasma and showed that the inflammatory z-score was elevated by the baked chow diet suggesting a pro-inflammatory effect. However, none of the inflammatory biomarkers included in this score varied with diet when measured individually. Next to mice studies, several human intervention trials have been performed to link the consumption of dietary AGEs to human health and disease. Since tissue accessibility in humans is limited, results are mainly directed toward indirect measures such as systemic oxidative stress and inflammation. Collectively these studies claimed that long-term restriction of dAGE consumption could improve health [[17], [18], [19], [20]]. However, since they prepared low- and high-AGE diets using different cooking techniques, other variables could have been affected as well, for example caloric density, micronutrient content, and formation of other Maillard Reaction Products [21]. Recently, an intervention study has investigated diets different in AGE content without them being prepared via different cooking methods. Instead, the intervention diets were composed of different food items, varying in AGE levels, while being energy- and macronutrient-matched. In contrast to the previous intervention studies, they did not observe differences between the diets [22]. Thus, whether the absorption of dietary AGEs, like the formation of endogenous AGEs, is part of disease pathologies is still under debate and a critical evaluation of the experimental design should be performed before drawing dietary recommendations.

## 3 Interaction of dietary protein-bound AGEs with the intestinal epithelium

Besides potentially exerting these systemic health effects after absorption, dietary AGEs might also interact with the intestinal epithelial cells directly. These cells are chronically exposed to relatively high levels of glycated proteins [13] that are hypothesized to exert a pro-inflammatory effect via interaction with membrane-bound receptors, mainly RAGE [23]. Tessier et al. [24] employed [^13^C_2_]-glyoxylic acid glycated bovine serum albumin-fed mice, showing that CML was accumulated at a high deposition rate in the ileum and colon. They showed that this accumulation was RAGE-independent. Unfortunately, due to the limited amount of tissue available, the authors were not able to qualify the CML accumulation in the tissue in terms of free versus protein-bound and intra-versus extracellular, nor could they access the physiological effects of this accumulation. Therefore, it is difficult to formulate a statement on the consequences of and mechanisms underlying tissue deposition of CML. Since glycated proteins are considered hardly bioavailable, the formation of free AGEs and dipeptides upon partial digestion of these proteins is potentially involved as they are described to be (partially) bioavailable, either via passive diffusion, paracellular transport or absorption via for example PEPT1 [14,25]. In theory, endocytic uptake and intracellular degradation of undigested glycated proteins and large peptides could also be involved [26]. However, this has mainly been shown in conditions of cellular stress when cells use the protein as a source of amino acids and energy [27]. Overall, protein-bound dietary AGEs may have other (indirect) modes of action than solely acting via membrane-bound receptors like RAGE, possibly also dependent on the inflammatory state.

## 4 Animal studies on dietary protein-bound AGEs and the intestinal epithelium

Human intervention studies have yet to be performed in this field. Nevertheless, a few *in vivo* animal studies investigated the effect of glycated proteins on the intestinal epithelium as a (secondary) outcome measure (see Table 1). Except for one study, they all used healthy rodent models exposed to high levels of AGEs, either by heating the diet or supplementing it with glycated proteins. Increasing the exposure to AGEs by heating the diet has some disadvantages, due to the introduction of uncontrolled variables. For example, micronutrient content may change, caloric density increases due to water loss, and other (possibly toxic) compounds unrelated to AGEs are formed. The study of Qu et al. [28] kept these limitations to a minimum by adjusting for the water loss and adding vitamins and minerals into the pellet only after the heat treatment. In addition, they quantified both free and protein-bound CML in the food pellets after heating with advanced mass spectrometry techniques. In this way the effectiveness of the glycation protocol was checked and dose comparison between studies was made possible. This analysis showed that most dietary AGEs formed were protein-bound. The authors observed that the histomorphology score of the colon deteriorated with intervention time, affecting the intestinal architecture and distorting intestinal crypts, depleting Goblet cells, and making cellular arrangements looser. However, tissue inflammation was not evident. In addition, the expression of the tight junction proteins ZO-1 and Occludin in the colonic epithelium was decreased after 18 weeks of intervention. This decreased expression of tight junction proteins could be indicative of the development of a leaky gut, which was also suggested by a modest increase of plasma LPS levels. Unfortunately, no direct measurement of gut permeability, for instance, by the FITC-Dextran method, was performed. In line with these results, Cao et al. [29] observed a reduced gene expression of the same tight junction proteins in colonic tissue after increasing the consumption of AGEs in mice by adding extensively glycated fish protein (dry glycation, 55 °C, 24 h) to the diet. In addition, this study showed increased colonic gene expression of RAGE, but did not find an effect on pro-inflammatory cytokines in the colonic tissue. When they supplemented the mice's diet with lightly or moderately glycated fish protein (dry glycation, 55 °C, resp. 6 and 12 h), these effects were not observed or observed in a reverse direction. Together, these two studies suggest that increased consumption of AGEs leads to increased intestinal permeability independent of inflammation but dependent on the degree of protein glycation. In comparison, two studies using healthy rodent models increased the AGEs intake only by heating the diet without controlling the other variables. They showed varying effects on the gene expression of multiple tight junction proteins in the jejunum and ileum [30] and no effect on colonic inflammation in terms of mast cell infiltration and the neutrophil marker myeloperoxidase (MPO) activity [31]. Next to this healthy model, Al Amir et al. [31] also looked at a DSS-induced colitis model, in which he observed less mast cell infiltration and lower MPO activity when mice were pre-exposed to an AGEs-rich diet. These intervention effects increased with prolonged heating of the diet. Overall, the current *in vivo* studies suggest that consumption of glycated proteins affects the intestinal epithelium differently depending on their degree of glycation and the inflammatory state of the intestine. However, the amount of data is limited and results should be verified in various models.

**Table 1:** A summary of in vivo animal studies published on the effect of glycated proteins on the intestinal epithelium.

*In Vivo* Animal Studies													
Model	Synthesis conditions				Characterization	Experimental conditions					Observed Effects		Reference
	Heating	Reducing Agent	Temperature	Time		Background Animal	Disease	Type of treatment	Duration	Control group	Tight Junctions	Inflammation	
Heated diet	Dry	N.A.	160 °C	1 h	NBT assay LC-MS/MS^a^	C57BL/6 mice	None	Standard diet	24 weeks	Non-heated diet^f^	↕	X	[30]
	Wet	N.A.	150 °C	1.5 h	None	Balb/c mice	None	Standard diet	3 weeks	Non-heated diet	X	–	[31]
	Wet	N.A.	150 °C	4 h	None	Balb/c mice	None	Standard diet	3 weeks	Non-heated diet	X	–	[31]
	Wet	N.A.	150 °C	1.5 h	None	Balb/c mice	DSS-induced colitis	Standard diet	3 weeks	Non-heated diet	X	↓	[31]
	Wet	N.A.	150 °C	4 h	None	Balb/c mice	DSS-induced colitis	Standard diet	3 weeks	Non-heated diet	X	↓	[31]
	Dry	N.A.	125 °C	3 h	LC-MS/MS^b^	Sprague–Dawley rats	None	Standard diet	18 weeks	Non-heated diet^g^	↓	X	[28]
Glycated protein	Dry	Glucose	55 °C	6 h	Fluorescent intensity LC-MS/MS^c^	C57BL/6 mice	None	Fish Protein	15 weeks	Non-heated fish protein with glucose^h^	↑	↓	[29]
	Dry	Glucose	55 °C	12 h	Fluorescent intensity LC-MS/MS^d^	C57BL/6 mice	None	Fish Protein	15 weeks	Non-heated fish protein with glucose^h^	–	–	[29]
	Dry	Glucose	55 °C	24 h	Fluorescent intensity LC-MS/MS^e^	C57BL/6 mice	None	Fish Protein	15 weeks	Non-heated fish protein with glucose^h^	↓	–	[29]

↑ = upregulated, ↓ = downregulated, ↕ = ambiguous, − = no effect, X = not investigated.

a 4.87 μg CML/g chow, 1.38 μg CEL/g chow, 43.49 μg MG-H1/g chow.

b 39 ng free CML/g chow, 14.45 μg protein-bound CML/g chow.

c 21 μg Furosine/mg protein, 8.29 ng CML/mg protein, 0.54 ng CEL/mg protein.

d 26.2 μg Furosine/mg protein, 12.76 ng CML/mg protein, 1.88 ng CEL/mg protein.

e 2.84 μg Furosine/mg protein, 13.31 ng CML/mg protein, 3.96 ng CEL/mg protein.

f 2.58 μg CML/g chow, 0.89 μg CEL/g chow, 34.51 μg MG-H1/g chow.

g 5 ng free CML/g chow, 2.79 μg protein-bound CML/g chow.

h 0.6 μg Furosine/mg protein, 1.05 ng CML/mg protein, 0.24 ng CEL/mg protein.

**Figure 1:**
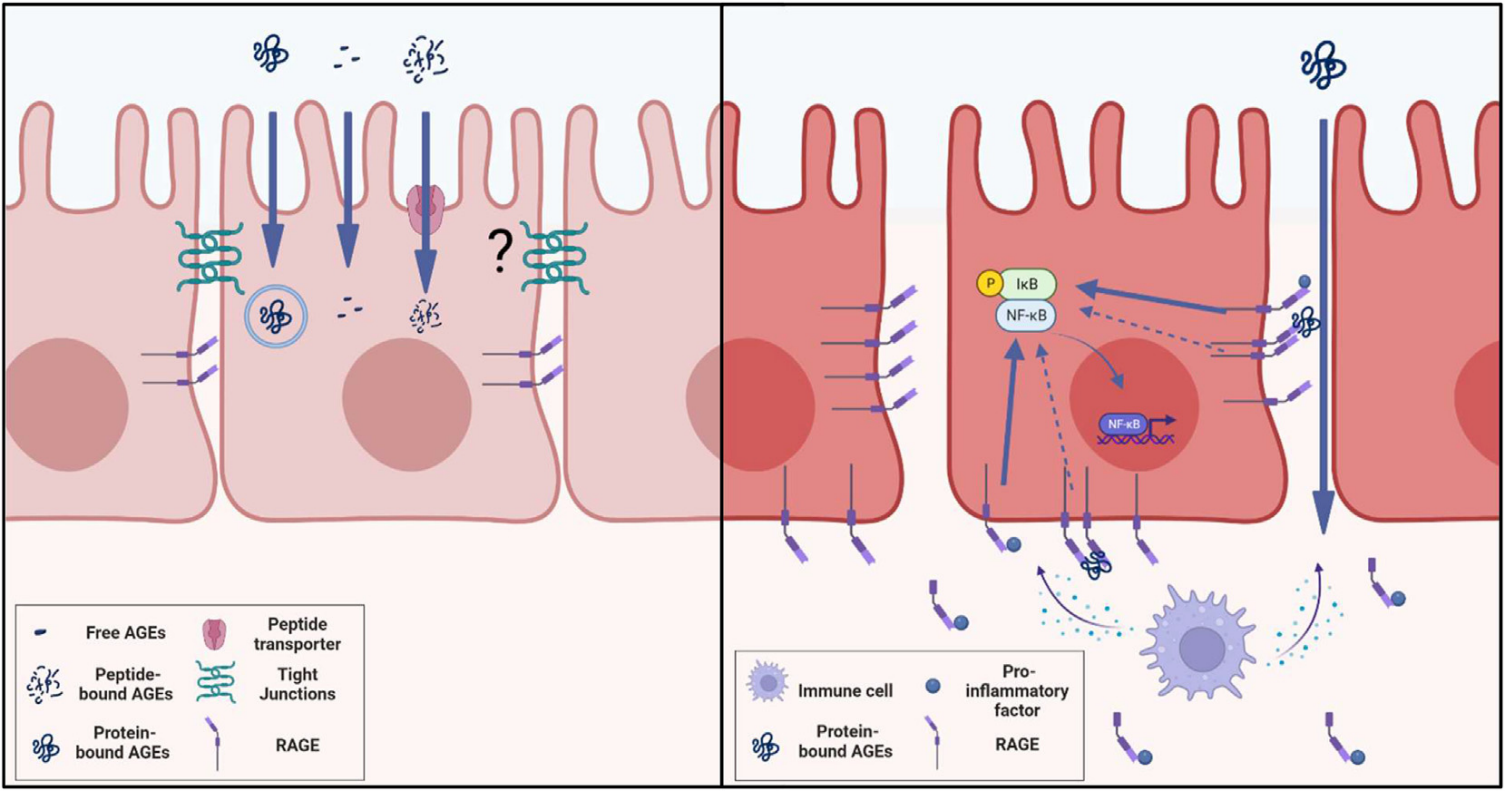
***Hypothesis on how dietary Advanced Glycation End products (AGEs) affect intestinal epithelial cells under homeostatic (left panel) and inflammatory conditions (right panel), based on results of in vivo rodent studies.*** Under homeostatic conditions, dietary AGEs seem to increase intestinal permeability independent from inflammation, while RAGE is expressed on the lateral side of epithelial cells underneath the tight junctions. Since especially protein-bound AGEs have affinity for RAGE, it is unlikely that this effect is induced in a RAGE-dependent manner as cellular arrangements under homeostatic conditions are too tight for proteins to pass the barrier paracellularly. It is more likely that the effect is induced by intracellular signaling of AGEs, being absorbed via passive diffusion, active transport or endocytosis. In case of inflammation, the intestinal barrier function is hampered. Therefore, in addition to the intracellular signaling mentioned above, protein-bound AGEs would be able to interact with RAGE, which is expressed on lateral as well as basolateral side under these conditions. Although data is limited, AGEs seem to have a protective effect on inflammation, suggesting that they would act as a partial agonist or antagonist on RAGE for NFκB activation or enhance scavenging or RAGE agonists by exaggerating RAGE shedding. Figure created with BioRender.com.

Overall, data from animal studies suggests that in healthy models dietary AGEs mainly affect tight junction expression and thus intestinal barrier integrity without directly inducing inflammation, while in a model for inflammatory bowel disease they showed a protective effect against inflammation (see Figure 1). Under healthy conditions, RAGE is expressed on the lateral side of epithelial cells beneath the tight junctions [32] and paracellular transport of protein-bound AGEs is unlikely since the space between epithelial cells is too narrow [33]. Since especially protein-bound AGEs have affinity for RAGE, the proposed effect of dietary AGEs on tight junction expression and gut barrier integrity is probably RAGE-independent. The effects are more likely to be caused by intracellular signaling of AGEs being absorbed through passive diffusion, active transport, or endocytosis [14,25,26]. Under inflammatory conditions, RAGE is also expressed on the basolateral membrane intestinal and intestinal permeability is increased [32], wherefore bigger proteins could pass the barrier paracellularly and bind RAGE. Treatment with a RAGE-specific inhibitor has been shown to protect mice from DSS-induced colitis [34], so the protective effect of dietary AGEs shown by Al Amir et al. [31] might be mediated by these compounds acting as a partial agonist or even antagonist to RAGE. On the other hand, dietary AGEs may act anti-inflammatory through stimulating RAGE shedding. RAGE shedding has been observed in active IBD, leading to increased levels of sRAGE [35]. As sRAGE scavenges RAGE agonists, dietary AGEs exaggerating the RAGE shedding observed in IBD might further dampen RAGE activation. In conclusion, the impact of dietary AGEs on intestinal epithelial cells might deviate between healthy and diseased models and might depend on the size of the AGEs and whether enterocytes can internalize them.

## *5* *In vitro* studies on dietary protein-bound AGEs and the intestinal epithelium

Next to the rodent studies, i*n vitro* studies have been performed to understand better the mechanisms behind the effects of glycated proteins on the intestinal epithelium (Table 2). Different model proteins have been used: mainly bovine serum albumin (BSA) but also peanut 7 S globulin and casein. Studies investigating the glycation of casein primarily focused on its potential pro-inflammatory properties. Jing et al. [36] showed that glycated casein decreased proliferation and antioxidant enzyme activity in an Int-407 *in vitro* model, but not in a Caco-2 model. Comparing these cell lines, Int-407 cells were considered healthy since they are cultured from normal embryonic intestinal tissue, whereas Caco-2 cells originate from a colon carcinoma. Later on, Int-407 was shown to be derived by HeLa contamination [37], so strictly, it cannot be considered a healthy intestinal model anymore. Another study used a cell line cloned from Caco-2, C2BBe1, and reported that glycated casein increased proliferation, oxidative stress, and IL-8 secretion compared to native casein [38]. In this polarized monolayer, activation of the pro-inflammatory NFkB and Akt-mTORC signaling pathways was also observed. These inconsistent results about the inflammatory properties of glycated proteins can be due to different protein aggregation patterns and AGE levels. Aggregated proteins are described to have an increased affinity for the RAGE receptor [39,40], however, aggregation can be caused by heat-induced protein unfolding as well as glycation-dependent protein cross-linking [41]. It has not yet been described whether RAGE affinity and epithelial responses depend on the nature of aggregation, i.e. AGEs being present or absent. Therefore, future studies focussing on the inflammatory properties of glycated proteins on the intestinal epithelium should be conducted after a detailed physicochemical characterization of the glycated protein. In addition, when studying the inflammatory properties of biological macromolecules, it is important to check for possible endotoxin contamination in the sample as this can interfere with the measurement. Unfortunately, in general, this confounder is often overlooked.

**Table 2:** A summary of in vitro studies published on the effect of glycated proteins on intestinal epithelial cells.

*In Vitro* Studies															
Protein	Synthesis conditions				Characterization	Cell line	Exposure conditions		Controls			Observed effects			Reference
	Heating	Reducing Agent	Temperature	Time			Concentration of protein	Time	Medium	Native	Heated	Proliferation	Inflammation/Oxidative stress	Cancer promotion	
Bovine Serum Albumin	Wet	Glucose	37 °C	8 weeks	Fluorescent intensity	HCT116	50 μg/mL	12–48 h	No	No	Yes	–	X	**↑**	[42]
	Wet	Glyceraldehyde	37 °C	10 days	None	HCT116	200 μg/mL	24 h	No	No	Yes	X	X	**↑**	[43]
	Wet	Glucose	37 °C	8 weeks	None	HCT116	200 μg/mL	24 h	No	No	Yes	**↑**	X	**↑**	[44]
	Wet	Glucose	37 °C	8 weeks	None	SW620	200 μg/mL	72 h	No	No	Yes	X	X	**↑**	[45]
	Wet	Glucose	37 °C	8 weeks	None	LoVo	200 μg/mL	72 h	No	No	Yes	X	X	**↑**	[45]
Casein	Wet	Glucose/Fructose	55 °C	18 days	Colorimetric index Available lysine	Caco-2	0.5–2.0 mg/mL	3 h	Yes	Yes	No	**−**	**−**	X	[36]
	Wet	Glucose/Fructose	55 °C	18 days	Colorimetric index Available lysine	Int-407	0.5–2.0 mg/mL	3 h	Yes	Yes	No	**↓**	**↓**	X	[36]
	Wet	Mixture of glucose, fructose and lactose	70 °C^d^	30 min	LC-MS/MS^a^ Size exclusion chromatography Fluorescent intensity ELISA	C2BBe1	200 μg/mL	3–24 h	No	Yes^b^	No	**↑**	**↑**	X	[38]
Peanut 7 S globulin	Dry	Glucose	37 °C	7 days	OPA assay SDS-PAGE Colorimetric index Fluorescent intensity	Caco-2	500 μg/mL (proliferation)/25 μg/mL (inflammation)	Resp. 1.5 and 24 h	Yes	Yes	Yes^c^	**−**	**↓**	X	[47]
	Dry	Glucose	60 °C	3 days	OPA assay SDS-PAGE Colorimetric index Fluorescent intensity	Caco-2	500 μg/mL (proliferation)/25 μg/mL (inflammation)	Resp. 1.5 and 24 h	Yes	Yes	Yes^c^	**↑**	**↓**	X	[47]
	Dry	Glucose	145 °C	20 min	OPA assay SDS-PAGE Colorimetric index Fluorescent intensity	Caco-2	500 μg/mL (proliferation)/25 μg/mL (inflammation)	Resp. 1.5 and 24 h	Yes	Yes	Yes^c^	**↑**	**↓**	X	[47]

↑ = upregulated, ↓ = downregulated, ↕ = ambiguous, − = no effect, X = not investigated.

a 267 μg total AGEs/mg protein.

b 11 μg total AGEs/mg protein.

c Outcomes compared to heated controls.

d Mimicking ultra-high-temperature processing of milk: 70 °C for 30min, followed by 135 °C for 8 s and stored for 90 days at 25 °C.

Next to pro-inflammatory properties, potential cancer-promoting effects of glycated proteins have been studies as well [[42], [43], [44], [45]]. They all used BSA as model protein and colon-cancer-derived immortalized cell lines expressing relatively high levels of RAGE (HCT116, SW620, and LoVo) [45]. Three of these four studies showed that observed cancer-promoting effects were (partially) RAGE-dependent [42,44,45]. In this respect, Wang et al. [42] described RAGE-dependent upregulation of the oncogene MDM2 and inactivation of related tumor suppressors p53 and Rb, while Lin et al. [43] showed increased translocation of β-catenin, which has been described to promote transcription of several oncoproteins [46], accompanied with the upregulation of RAGE. In addition, H. Chen et al. [44] and R. Deng et al. [45] showed a RAGE-dependent increase of the expression of proteins involved in respectively cell proliferation (ChREBP) and cancer cell migration (Sp1 and MMP2). Related to these outcomes, these studies also showed increased cell proliferation and increased invasion and migration rates, respectively. So, overall, these studies suggest cancer-promoting properties of glycated BSA, however, their methodology faces some limitations. For example, the studies used heated BSA as a negative control, while preferably a native BSA and medium control would be included as well since Teodorowicz et al. [47] showed that glycated protein increased proliferation (and decreased inflammation) compared to the native and heated protein, while there was no or an opposite effect on these parameters compared to the medium control, when investigating the effect of glycated peanut 7 S globulin on a Caco-2 model. In addition, these studies did not always check for dose-dependency, which would prove causality, nor did they include a compound known to promote cancer, preferably through RAGE, as a positive control. Therefore, it is hard to make a statement about the potency of glycated BSA in inducing the observed effect. So, although evidence seems to suggest that glycated BSA has a cancer-promoting effect in immortalized cancer colon-derived cell lines, the results should be interpreted with caution.

## 6 Studies on dietary free AGEs and the intestinal epithelium

The abovementioned studies investigated dietary protein-bound AGEs. In general, this research focused on their effects on the colonic epithelium, related to the reduced digestibility and bioavailability of these proteins shown *in vitro*. Apart from this, some *in vivo* and *in vitro* studies investigated the effects of free AGEs, mainly free CML, in Caco-2 cells [[48], [49], [50]] and rodent models [51]. For the *in vitro* studies, Holik et al. [49] and Wu et al. [50] differentiated the Caco-2 cells to produce a small intestinal-like phenotype, while Z. Chen et al. [48] did not differentiate the cells. Wu et al. [50] showed increased ROS generation, MAPK and NFkB protein expression, and decreased ZO-1 protein expression, while Holik et al. [43] reported no effect on proliferation. In comparison, Z. Chen et al. [48] observed increased proliferation but exposed the cells to 20 mM free CML which is a 40-times higher concentration than tested by Holik et al. [49] and Wu et al. [50]. *In vivo*, potential pro-inflammatory effects of free CML, as suggested by the *in vitro* study of Wu et al. [50], were not observed in a healthy nor in a colitis mouse model [51]. Since free CML was shown to lack affinity for RAGE [39] but can enter the cells by passive diffusion [52], its potential cellular effects are more likely to be initiated by intracellular interactions with proteins and/or receptors. In this case, the mTOR pathway may be a potential candidate since it stimulates proliferation in the presence of amino acids [53]. However, current literature is not conclusive as to which pathways and processes might be affected by free AGEs in the intestinal epithelium.

## 7 Concluding remarks and future research

Taken together, *in vitro* studies are a valuable tool to understand underlying mechanisms of observations made *in vivo*, however the design of the currently available *in vitro* studies face some limitations and should therefore be interpreted with caution. Future *in vitro* studies focusing on the biological properties of dietary AGEs should only be conducted after a detailed physicochemical characterization of the glycated protein and the quantification of endotoxins in the sample. If endotoxins are detected, it has to be confirmed that the *in vitro* model of interest is not affected by their presence, otherwise the endotoxins in the sample need to be removed or neutralized before conducting any experiment on this model. In addition, based on its research question and hypothesis, future studies should consider carefully which negative and positive controls to include. When glycating a protein of interest, reducing sugars react with free amino groups of the target protein under the influence of heat. So, for the negative controls, future studies should not only control for the effect of a reducing sugar modifying the protein by including a heated protein control, but also for the effect of heating a protein and the presence of the protein itself, respectively by including a native protein and medium control. Besides, especially when the reducing sugar used is highly potent, the sample needs to be checked for any residues of this reactant as this could affect the cells. Considering the positive controls, a compound already known to induce the hypothesized outcome must be included. When this outcome is expected to be RAGE dependent, the positive control should also be described in the literature to act through RAGE. Lastly, dose-dependency between exposure and outcome must be shown to prove causality. In summary, since the glycation process introduces many additional variables and the generated AGEs are a heterogeneous group of compounds, it is of paramount importance to characterize the glycated protein thoroughly and to check and control for all possible confounders that might be introduced in the *in vitro* model.

Until now, all these *in vitro* studies use immortalized cell lines from colon cancer origin, mainly Caco-2. Although these cells are widely used and accepted as a model for intestinal epithelial cells, it cannot be excluded that they might respond differently from healthy epithelial cells [54], for example when RAGE appears to be differently expressed on or distributed over the cell membrane. Therefore, using intestinal organoids as a model could be the next step to increase the external validity of *in vitro* studies. In addition, the interaction between dAGEs and the gut microbiota should be considered as a variable to control, as dAGEs are subject to microbial fermentation [55,56]. As gut microbial metabolites are known to interact with the intestinal epithelium and exert immunomodulatory properties, exposure to gut microbial metabolites derived from the fermentation of AGEs and co-cultures with immune cells would be necessary to cover the interplay between epithelial cells, the microbiome, and mucosal immune system [57]. By operating a more advanced *in vitro* model, such studies could be more effectively translated to human studies limiting the use of experimental animals as much as possible.

## Declaration of Competing Interest

The authors declare that they have no known competing financial interests or personal relationships that could have appeared to influence the work reported in this paper.

## Data Availability

No data was used for the research described in the article.
